# Variable Sensitivity of SARS-CoV-2 Molecular Detection in European Expert Laboratories: External Quality Assessment, June and July 2020

**DOI:** 10.1128/JCM.02676-20

**Published:** 2021-02-18

**Authors:** Carlo Fischer, Ramona Mögling, Angeliki Melidou, Arne Kühne, Edmilson F. Oliveira-Filho, Thorsten Wolff, Janine Reiche, Eeva Broberg, Christian Drosten, Adam Meijer, Katrin Leitmeyer, Jan Felix Drexler, Chantal B. E. M. Reusken

**Affiliations:** a Charité-Universitätsmedizin Berlin, corporate member of Freie Universität Berlin, Humbolt-Universität zu Berlin and Berlin Institute of Health, Institute of Virology, Berlin, Germany; b Centre for Infectious Disease Control, National Institute for Public Health and the Environment (RIVM), Bilthoven, the Netherlands; c European Centre for Disease Prevention and Control (ECDC), Stockholm, Sweden; d Robert Koch Institute, Berlin, Germany; e German Centre for Infectious Diseases (DZIF), associated partner site Charité, Berlin, Germany; Cepheid

**Keywords:** SARS-CoV-2, COVID-19, EQA, molecular, diagnostics, Europe, SARS-CoV-2

## Abstract

During the ongoing coronavirus disease 2019 (COVID-19) outbreak, robust detection of severe acute respiratory syndrome coronavirus 2 (SARS-CoV-2) is a key element for clinical management and to interrupt transmission chains. We organized an external quality assessment (EQA) of molecular detection of SARS-CoV-2 for European expert laboratories. An EQA panel composed of 12 samples, containing either SARS-CoV-2 at different concentrations to evaluate sensitivity or other respiratory viruses to evaluate specificity of SARS-CoV-2 testing, was distributed to 68 laboratories in 35 countries. Specificity samples included seasonal human coronaviruses hCoV-229E, hCoV-NL63, and hCoV-OC43, as well as Middle East respiratory syndrome coronavirus (MERS-CoV), SARS-CoV, and human influenza viruses A and B. Sensitivity results differed among laboratories, particularly for low-concentration SARS-CoV-2 samples. Results indicated that performance was mostly independent of the selection of specific extraction or PCR methods.

## Abstract (precis)

During the ongoing coronavirus disease 2019 (COVID-19) outbreak, robust detection of severe acute respiratory syndrome coronavirus 2 (SARS-CoV-2) is a key element for clinical management and to interrupt transmission chains. We organized an external quality assessment (EQA) of molecular detection of SARS-CoV-2 for European expert laboratories. An EQA panel composed of 12 samples, containing either SARS-CoV-2 at different concentrations to evaluate sensitivity or other respiratory viruses to evaluate specificity of SARS-CoV-2 testing, was distributed to 68 laboratories in 35 countries.

## INTRODUCTION

As of December 2020, severe acute respiratory syndrome coronavirus 2 (SARS-CoV-2) has infected over 66 million individuals and caused more than 1,500,000 deaths globally (https://covid19.who.int/; accessed 7 December 2020). As both specific medications and approved vaccines are not available yet, public health strategies need to focus on containment and mitigation measures. Robust detection of acute SARS-CoV-2-infected individuals, typically done by real-time reverse transcription PCR (rRT-PCR), is crucial for clinical management, surveillance and to interrupt transmission chains (1). An established tool to improve and support diagnostic accuracy for clinical management and surveillance use is the conduction of external quality assessments (EQA) (2–5). An EQA of molecular detection of SARS-CoV-2 was organized for the expert laboratories of the Emerging Viral Diseases–Expert Laboratory Network (EVD-LabNet) and/or the European Reference Laboratory Network for Human Influenza (ERLI-Net).

## MATERIALS AND METHODS

### Panel composition.

The EQA panel was composed of 12 samples containing either SARS-CoV-2 at different concentrations to evaluate sensitivity or other respiratory viruses to evaluate specificity of SARS-CoV-2 testing (Table 1).

**TABLE 1:** EQA panel composition and correct test results of participating laboratories

Sample ID	Virus^*a*^	No. of copies/µl	Correct results^*b*^	
			%	No./total
1	SARS-CoV-2	2	60	41/68
2	SARS-CoV-2	12.5	99	67/68
3	HMPV A	500	96	65/68
	HMPV B	2,000	96	65/68
	PIV1	2,000	96	65/68
	PIV2	2,000	96	65/68
	PIV3	2,000	96	65/68
	PIV4	2,000	96	65/68
	RSV	1,500	96	65/68
4	MERS-CoV	5,000	98	65/66
5	*Enterovirus B* (Echovirus 6)	2,000	100	68/68
	*Rhinovirus* (Tu-12-1331 HRV A)	1,000	100	68/68
	*Influenzavirus A*	2,000	100	68/68
	H1N1 pdm09	500	100	68/68
	*Influenzavirus B*	2,000	100	68/68
	*Adenovirus*	2,000	100	68/68
6	SARS-CoV-2	2	60	41/68
7	hCoV-229E	1,000	97	66/68
	hCoV-NL63	2,000	97	66/68
8	hCoV-OC43	2,000	97	66/68
9	SARS-CoV	5,000	89	59/66
10	SARS-CoV-2	12.5	90	61/68
11	SARS-CoV-2	2,500	99	67/68
12	SARS-CoV-2	180	99	67/68

a Enterovirus, rhinovirus, respiratory syncytial virus (RSV), adenovirus, influenza A virus H1N1, influenza B virus, middle east respiratory syndrome coronavirus (MERS-CoV), severe acute respiratory syndrome coronavirus (SARS-CoV), and severe acute respiratory syndrome coronavirus 2 (SARS-CoV-2) BetaCoV/Munich/ChVir984/2020 were grown on Vero cells. Parainfluenza virus 1 (PIV1), parainfluenza virus 2 (PIV2), parainfluenza virus 3 (PIV3), parainfluenza virus 4 (PIV4), human coronavirus 229E (hCoV-229E), and human coronavirus OC43 (hCoV-OC43) were grown on CaCo-2 cells. Human metapneumovirus A (hMPV A), human metapneumovirus B (hMPV B), and human coronavirus NL63 (hCoV-NL63) were grown on LLC-MK2 cells.

b Laboratories were not asked to identify the specific viruses, only to indicate whether the samples were SARS-CoV-2 positive or not.

### Specimen preparation.

SARS-CoV-2 samples were quantified using an E-gene-based in-house assay recommended by the WHO (6) that relies on an *in vitro*-transcribed RNA standard. Samples were diluted in Dulbecco’s modified Eagle’s medium (DMEM). All samples were heat-inactivated following Matheeussen et al. (4) (65°C for 4 h for SARS-CoV, SARS-CoV-2, MERS-CoV, and adenovirus, and for 2 h for all other viruses listed in Table 1), and 200 µl of each sample was freeze-dried.

### Panel validation, testing instructions, and panel dispatch.

Successful inactivation of panel samples was confirmed by the absence of viral growth in three consecutive cell culture passages. Along with the EQA panels, participants received detailed reconstitution and testing instructions. The panels were shipped at ambient temperature. If national legislation of a country prohibited receipt of inactivated SARS-CoV and MERS-CoV samples, the laboratories received a modified panel that excluded these sample(s). Two independent laboratories validated the composition of the EQA panel and the result submission platform before distribution to all other EQA participants.

### Evaluation of results.

Result reporting was done via an online submission form by which laboratories could score their samples (positive, negative, or inconclusive) and provide information such as cycle threshold (*C_T_*) values, extraction methods and type of PCR(s). Laboratories were asked to treat and evaluate the EQA samples according to their routine diagnostic workflow, which could include the use of multiple assays. To ensure uniform interpretation, a sample was scored as positive if at least one SARS-CoV-2 test gave a positive result. No *C_T_* threshold was applied.

Sample no. 9, containing SARS-CoV, was not considered a “core sample,” and was therefore not included in the laboratory performance analysis. Core samples are defined as clinically relevant samples for proficiency testing. Clinical relevancy was given for the detection of all SARS-CoV-2 samples and for distinction of SARS-CoV-2 samples from other human respiratory viruses that are circulating in the human population. Since SARS-CoV has not been circulating in the human population since 2004, the capability to distinguish between SARS-CoV and SARS-CoV-2, which are closely related, is informative but unnecessary (7).

EQA participants were only asked to report whether samples were SARS-CoV-2 positive or not.

### Statistical analysis.

Statistical analyses were conducted to examine if the overall EQA performance correlated with specific technical details provided by the EQA participants. The information about workflows and protocols provided by the EQA participants was reviewed to be conclusive regarding applied kits or tests. If needed, participants were contacted to confirm or specify the provided information. Nevertheless, the reported information was not complete for all participants. The utilized data sets did thus differ slightly in size among statistical analyses of different variables. Before conducting statistical analyses, variables were tested for normal distribution using the Shapiro-Wilk test. All statistical analyses were conducted in R version 4.0.2.

Considering the incompleteness of reported information and the high diversity of reported workflows (see Fig. S1 in the supplemental material), different steps of the diagnostic workflow were analyzed individually. For this purpose, each variable was analyzed statistically, including the results of all EQA participants that provided reliable information for it. For some variables, such as applied PCR assays, we both tested for a general trend of correlation between the variable and the number of correct results and compared the amounts of correct results between specifically selected fractions of the variable, e.g., comparing the results between two specific real-time RT-PCR assays.

## RESULTS

**FIG 1:**
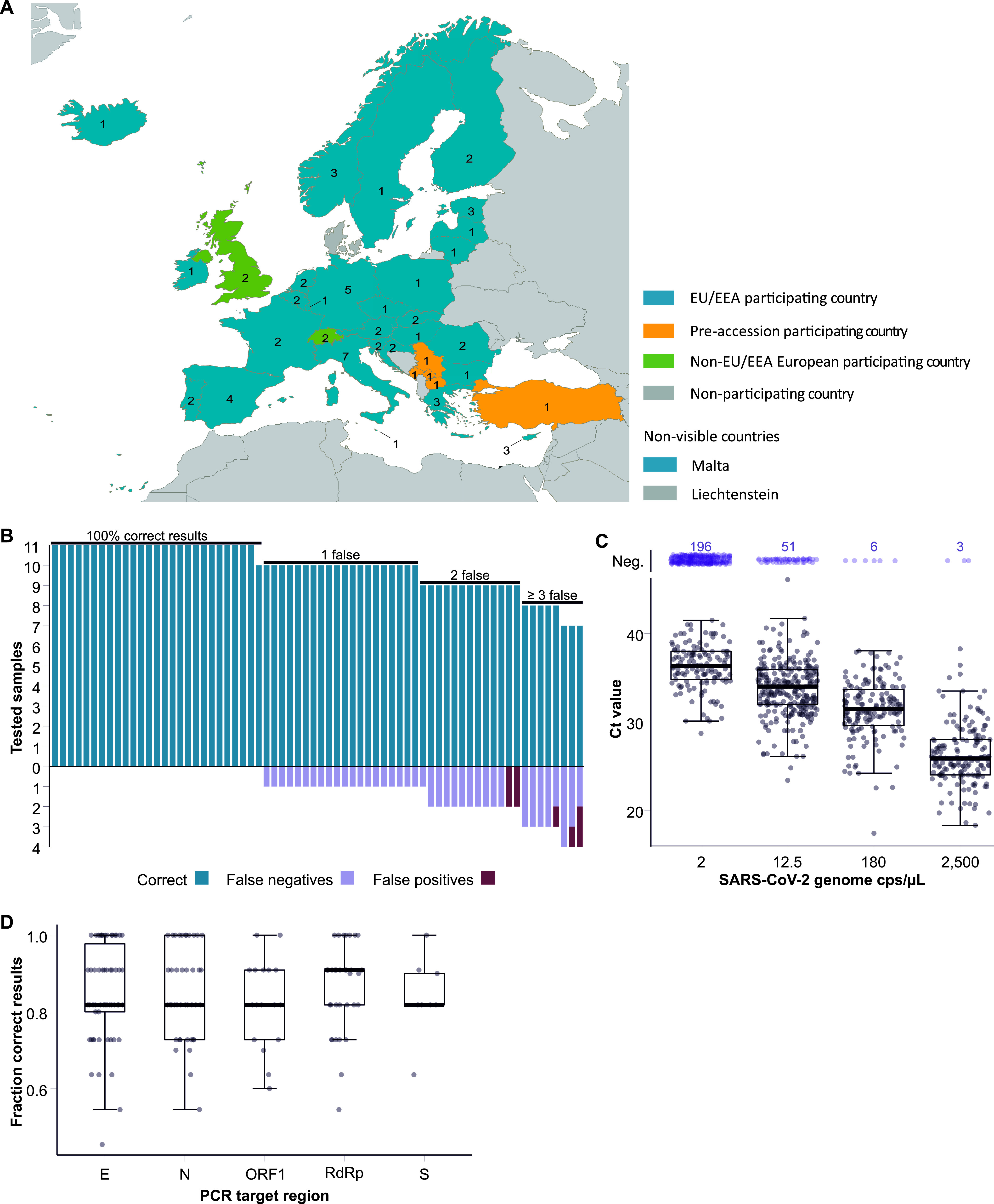
Participating laboratories in SARS-CoV-2 molecular EQA and laboratory performance in relation to specific factors. (A) Overview of countries of the participating laboratories in SARS-CoV-2 molecular EQA, June and July 2020. (B) Numbers of correct, false-negative, and false-positive samples per laboratory. (C) Reported *C_T_* values per SARS-CoV-2-positive sample considering all results reported, including multiple tests conducted by one participant. Median *C_T_* values are indicated by bars, quartiles by boxes, and interquartile range by whiskers. Median *C_T_* values were 25.9 (95% confidence interval [CI], 25.7 to 26.6) for sample 11 (2,500 copies [cps]/µl), 31.6 (95% CI, 31.1 to 32) for sample 12 (180 cps/µl), 34.0 (95% CI, 33.6 to 34.6) for samples 2 and 10 (12.5 cps/µl), and 36.4 (95% CI, 35.6 to 36.9) for samples 1 and 6 (2 cps/µl). Single events are indicated by gray (positive) and purple (negative) dots. (D) EQA performance, represented as a fraction of correct results (1.0 = 100% correct and 0.8 = 80% correct), depending on different rRT-PCR targets. For box plot explanation, see panel B.

Sixty-eight laboratories from 35 countries, i.e., 28 of 30 European Union/European Economic Area countries, five of seven European Union preaccession countries, and two other European countries, reported EQA results (Fig. 1A).

Twenty-seven of 68 participating laboratories tested all core samples correctly (Fig. 1B), 21 reported correct results for 10 samples, and 20 for nine samples or fewer. Notably, only 37 result submissions were based on the outcome of a single test method. The remaining participants scored their results based on the outcomes of two or more test methods. Incorrect results were reported for 9.7% (72/746) of all samples, with 8.6% (64/746) false-negative results reported for SARS-CoV-2 samples and 1.1% (8/746) false-positive results reported for specificity controls (Table 1). The risk of false-negative tests increased significantly with lower SARS-CoV-2 concentrations (*P* < 0.001; Spearman correlation test) (Fig. 1C). *C_T_* values of correctly tested SARS-CoV-2 samples increased with decreasing concentration (Fig. 1C). Sixty-three laboratories tested all specificity samples correctly, while 5 laboratories reported either one or two false-positive results. No single included respiratory virus was more prone to false-positive test results than others, pointing to contamination issues rather than issues intrinsic to the primer/probe sets used (Table 1).

As the performance in an EQA is the outcome of different steps in the diagnostic workflow combined, preferably different workflows should be compared. These steps comprise, e.g., resuspension of the panel samples, extraction method/kit used, change in nucleic acid (NA) concentration during extraction, and type of PCR assay, including the genomic target and the number of different genomic targets/tests performed. Among the participants of this EQA, there was an extensive variety of rRT-PCR methods that was used (Table 2; see also Table S2 in the supplemental material). The number of diagnostic workflows, which further included the extraction method/kit used and the RNA concentration change during extraction, was so high that a joint analysis of complete workflows was not possible (see Fig. S1 in the supplemental material), only allowing for analysis of individual components of the workflow.

**TABLE 2:** Performance of different rRT-PCR assays^*a*^

rRT-PCR assay (reference)	*N*^*b*^	Methodtype	Genome target	Assay performance (%) for:												EQA performance (core samples^*c*^)		
				Sample 11(SARS-CoV-2,2.50E+06 cps/ml)	Sample 12(SARS-CoV-2,1.80E+05 cps/ml)	Sample 2(SARS-CoV-2,1.25E+04 cps/ml)	Sample 10(SARS-CoV-2,1.25E+04 cps/ml)	Sample 1(SARS-CoV-2,2.00E+03 cps/ml)	Sample 6(SARS-CoV-2,2.00E+03 cps/ml)	Sample 7(hCoV-229Eand hCoV-NL63)	Sample 8(hCoV-OC43)	Sample 4(MERS-CoV)	Sample 3(respiratorypanel A)	Sample 5(respiratorypanel B)	Sample 9(SARS-CoV)	Total correct(%)	Falsepos. (%)	Falseneg. (%)
Corman et al. (6)	31	In-house	E, Sarbeco	100	100	97	87	35	39	97	100	100	94	100	100	86	2	24
Corman et al. (6)	11	In-house	RdRp, SARS-CoV-2	100	100	100	73	18	36	100	100	100	100	100	90	84	0	29
US CDC, USA (17)	10	In-house	N, SARS-CoV-2	100	100	90	70	60	70	100	100	100	100	100	100	90	0	18
Institute Pasteur, Paris, France (18)	10	In-house	RdRp, SARS-CoV-2	100	100	100	90	70	50	100	100	100	100	100	88	92	0	15
Allplex 2019-nCoV assay	9	Commercial	E, Sarbeco	100	89	89	56	44	56	100	100	100	100	100	67	85	0	28
Allplex 2019-nCoV assay	9	Commercial	N, SARS-CoV-2	100	100	78	89	56	56	100	100	89	100	100	100	88	2	20
Allplex 2019-nCoV assay	9	Commercial	RdRp, SARS-CoV-2	100	89	78	67	44	33	100	100	100	100	100	100	83	0	31
RealStar SARS-CoV-2 RT-PCR kit 1.0	5	Commercial	E, Sarbeco	100	100	100	100	40	40	100	100	100	100	100	100	89	0	20
RealStar SARS-CoV-2 RT-PCR kit 1.0	5	Commercial	S, SARS-CoV-2	100	100	100	100	20	40	100	100	100	100	100	100	87	0	23
Cobas SARS-CoV-2 test	5	Commercial	E, Sarbeco	80	100	100	60	40	40	100	100	100	100	100	80	84	0	30
Cobas SARS-CoV-2 test	5	Commercial	ORF1ab, SARS-CoV-2	80	100	100	60	80	40	100	100	100	100	100	100	87	0	23
Corman et al. (6)	5	In-house	N, Sarbeco	100	100	100	60	0	0	100	100	100	100	100	100	78	0	40
Viasure SARS-CoV-2 real-time PCR detection kit	5	Commercial	N, SARS-CoV-2	100	100	100	100	0	40	100	100	100	80	100	80	84	4	27
Viasure SARS-CoV-2 real-time PCR detection kit	5	Commercial	ORF1ab, SARS-CoV-2	100	80	100	100	0	0	100	100	100	100	100	100	80	0	37
Others	38	Both	Various	97	92	79	71	37	39	100	97	100	95	100	NA	82	6	31

a None of the assays performed significantly better than “Others” considering two-sided Yates’ corrected chi square. Sample 9 was excluded for calculation of the EQA performances.

b Number of EQA participants that performed the respective rRT-PCR assay.

c Clinically relevant samples that were used to assess laboratory performance in this study. Clinical relevance was given to SARS-CoV-2 samples of different concentrations and to samples containing other human respiratory viruses that are currently circulating in the human population. Sample no. 9 (SARS-CoV) is currently not circulating in the human population and therefore is not a core sample. pos., positive; neg., negative.

The performance among laboratories conducting manual NA extraction (92.4% correct tests) and laboratories conducting automated extraction (89.8% correct tests) did not differ significantly (*P* = 0.350; Mann-Whitney U test). The EQA performance was not correlated with the type of extraction kit used (*P* = 0.938; Kruskal-Wallis test) and ranged between 87.9% and 93.8% correct results (see Table S1 in the supplemental material). In most extraction protocols NA concentration is increased. However, the extent of NA concentration was not correlated with diagnostic sensitivity (*P* = 0.898; Spearman correlation test).

In total, 26 commercial assays and six in-house assays were used by the EQA participants. Among those assays performed by at least five EQA participants, correct results ranged between 78% and 92% (Table 2). The performance was not significantly correlated with a type of PCR assay in general (*P* = 0.525; Kruskal-Wallis test). However, results were significantly better with the best-performing test (Institute Pasteur, RdRp gene; Table 2) compared to “Others” (*P* = 0.042; Wilcoxon rank sum test), Corman N assay (*P* = 0.008), and the Viasure ORF1 assay (*P* = 0.011). The performances among commercial tests (84.6% correct results) and in-house tests (85.7% correct results) did not differ significantly (*P* = 0.969; Wilcoxon rank sum test). As transcription in coronaviruses typically varies among different genomic and subgenomic regions, target sites of the applied assays may affect the diagnostic sensitivity (8). However, the proportion of correct results did not differ significantly among different genomic targets (*P* = 0.852; Kruskal-Wallis test) (Fig. 1D).

## DISCUSSION

The overall performance within this EQA was variable. Most false results were reported for low-concentration SARS-CoV-2 samples. While infectious SARS-CoV-2 patients commonly have high viral titers around the first day of illness (9, 10) an optimized diagnostic sensitivity is important to identify patients outside the optimal window for detection or when sampling or transport were suboptimal (11). Low concentrations in clinical samples are also often seen at a later infection stage that coincides with seroconversion and a drop in infectivity (12). The inclusion of serological tests such as enzyme-linked immunosorbent assays in diagnostic algorithms when a diagnosis is needed for proper patient management and infection prevention measures, e.g., in hospitalized patients under strong suspicion of a SARS-CoV-2 infection but with repeated rRT-PCR-negative results, may reduce the risk of false-negative tests.

Ten laboratories showed problematic EQA performances, including five laboratories which had false-positive results. Notably, the laboratories reporting false-positive results applied both extraction kits and rRT-PCR tests also used by other laboratories. False-positive test results could thus be a consequence of contamination during sample handling and extraction or lot-specific contamination of rRT-PCR kits or oligonucleotides, as recently reported (13). Either way, laboratories should adapt workflows to ensure good specificity, which in general was excellent among participants.

Compared to the performance of first-line, routine clinical laboratories in a recently published SARS-CoV-2 EQA, the sensitivity of participants in our EQA was lower (4). However, comparing both EQAs is difficult, as sample quantification and preparation were done differently, in this study more precisely using DMEM instead of transport medium for sample preparation and quantifying concentrations with classic rRT-PCR instead of droplet digital PCR. Based on detection limits for commercial and in-house rRT-PCR tests (14), the participants in the recently published SARS-CoV-2 EQA (4) seem to overperform, which may indicate in reality slightly higher sample concentrations than those quantified by the authors before shipment. Both routine clinical laboratories and expert laboratories performed well, considering that suboptimal sensitivity in EQAs for molecular diagnostics of recently emerged viruses is not uncommon (2, 15, 16).

Notably, the results indicate that performance can be increased by harmonized workflows, rather than by the selection of specific extraction or rRT-PCR kits. None of the individual components of the workflow that were analyzed for this study were able to significantly influence the overall performance of a laboratory.

The conduction of follow-up EQAs will be essential to further support European laboratories to systematically improve and maintain diagnostic capabilities, while the need for a robust global detection capability requires global EQA programs.

## Supplementary Material

Supplemental file 1

Supplemental file 2

Supplemental file 3

## References

[1] Hellewell J, Abbott S, Gimma A, Bosse NI, Jarvis CI, Russell TW, Munday JD, Kucharski AJ, Edmunds WJ, Funk S, Eggo RM, Centre for the Mathematical Modelling of Infectious Diseases COVID-19 Working Group. 2020. Feasibility of controlling COVID-19 outbreaks by isolation of cases and contacts. Lancet Glob Health 8:e488–e496. doi:10.1016/S2214-109X(20)30074-7. 32119825 PMC7097845

[2] Fischer C, Pedroso C, Mendrone A, Jr, Bispo de Filippis AM, Vallinoto ACR, Ribeiro BM, Durigon EL, Marques ETA, Jr, Campos GS, Viana IFT, Levi JE, Scarpelli LC, Nogueira ML, Bastos MS, Souza NCS, Khouri R, Lira S, Komninakis SV, Baronti C, Charrel RN, Kummerer BM, Drosten C, Brites C, de Lamballerie X, Niedrig M, Netto EM, Drexler JF. 2018. External quality assessment for Zika virus molecular diagnostic testing, Brazil. Emerg Infect Dis 24:888–892. doi:10.3201/eid2405.171747. PMC5938781 29470164

[3] Reusken CB, Mogling R, Smit PW, Grunow R, Ippolito G, Di Caro A, Koopmans M. 2018. Status, quality and specific needs of Ebola virus diagnostic capacity and capability in laboratories of the two European preparedness laboratory networks EMERGE and EVD-LabNet. Euro Surveill 23(19):pii=17-00404. https://www.eurosurveillance.org/content/10.2807/1560-7917.ES.2018.23.19.17-00404. 10.2807/1560-7917.ES.2018.23.19.17-00404 PMC5954606 29766839

[4] Matheeussen V, Corman VM, Mantke McCulloch DO, Lammens E, Goossens C, Niemeyer H, Wallace D, Klapper PS, Niesters P, Drosten HG, Ieven C; on behalf of the RECOVER project and collaborating networks. 2020. International external quality assessment for SARS-CoV-2 molecular detection and survey on clinical laboratory preparedness during the COVID-19 pandemic, April/May 2020. Euro Surveill 25(27):pii=2001223. https://www.eurosurveillance.org/content/10.2807/1560-7917.ES.2020.25.27.2001223. 10.2807/1560-7917.ES.2020.25.27.2001223 PMC7364759 32672149

[5] Sung H, Han MG, Yoo CK, Lee SW, Chung YS, Park JS, Kim MN, Lee H, Hong KH, Seong MW, Lee K, Chun S, Lee WG, Kwon GC, Min WK. 2020. Nationwide external quality assessment of SARS-CoV-2 molecular testing, South Korea. Emerg Infect Dis 26:2353–2360. doi:10.3201/eid2610.202551. 32723432 PMC7510727

[6] Corman VM, Landt O, Kaiser M, Molenkamp R, Meijer A, Chu DK, Bleicker T, Brunink S, Schneider J, Schmidt ML, Mulders DG, Haagmans BL, van der Veer B, van den Brink S, Wijsman L, Goderski G, Romette JL, Ellis J, Zambon M, Peiris M, Goossens H, Reusken C, Koopmans MP, Drosten C. 2020. Detection of 2019 novel coronavirus (2019-nCoV) by real-time RT-PCR. Euro Surveill 25(3):pii=2000045. https://www.eurosurveillance.org/content/10.2807/1560-7917.ES.2020.25.3.2000045. 10.2807/1560-7917.ES.2020.25.3.2000045 PMC6988269 31992387

[7] Peeri NC, Shrestha N, Rahman MS, Zaki R, Tan Z, Bibi S, Baghbanzadeh M, Aghamohammadi N, Zhang W, Haque U. 2020. The SARS, MERS and novel coronavirus (COVID-19) epidemics, the newest and biggest global health threats: what lessons have we learned? Int J Epidemiol 49:717–726. doi:10.1093/ije/dyaa033. 32086938 PMC7197734

[8] Thi Nhu Thao T, Labroussaa F, Ebert N, V'Kovski P, Stalder H, Portmann J, Kelly J, Steiner S, Holwerda M, Kratzel A, Gultom M, Schmied K, Laloli L, Husser L, Wider M, Pfaender S, Hirt D, Cippa V, Crespo-Pomar S, Schroder S, Muth D, Niemeyer D, Corman VM, Muller MA, Drosten C, Dijkman R, Jores J, Thiel V. 2020. Rapid reconstruction of SARS-CoV-2 using a synthetic genomics platform. Nature 582:561–565. doi:10.1038/s41586-020-2294-9. 32365353

[9] La Scola B, Le Bideau M, Andreani J, Hoang VT, Grimaldier C, Colson P, Gautret P, Raoult D. 2020. Viral RNA load as determined by cell culture as a management tool for discharge of SARS-CoV-2 patients from infectious disease wards. Eur J Clin Microbiol Infect Dis 39:1059–1061. doi:10.1007/s10096-020-03913-9. 32342252 PMC7185831

[10] Kim SE, Jeong HS, Yu Y, Shin SU, Kim S, Oh TH, Kim UJ, Kang SJ, Jang HC, Jung SI, Park KH. 2020. Viral kinetics of SARS-CoV-2 in asymptomatic carriers and presymptomatic patients. Int J Infect Dis 95:441–443. doi:10.1016/j.ijid.2020.04.083. 32376309 PMC7196533

[11] Bergmans BJM, Reusken CBEM, Van Oudheusden AJG, Godeke G-J, Bonacic Marinovic AA, de Vries E, Kluiters-de Hingh YCM, Vingerhoets R, Berrevoets MAH, Verweij JJ, Nieman A-E, Reimerink J, Murk J-L, Swart AN. 2020. Declining SARS-CoV-2 PCR sensitivity with time and dependence on clinical features: consequences for control. medRxiv 2020.08.23.20179408. doi:10.1101/2020.08.23.20179408.

[12] Wolfel R, Corman VM, Guggemos W, Seilmaier M, Zange S, Muller MA, Niemeyer D, Jones TC, Vollmar P, Rothe C, Hoelscher M, Bleicker T, Brunink S, Schneider J, Ehmann R, Zwirglmaier K, Drosten C, Wendtner C. 2020. Virological assessment of hospitalized patients with COVID-2019. Nature 581:465–469. doi:10.1038/s41586-020-2196-x. 32235945

[13] Mogling R, Meijer A, Berginc N, Bruisten S, Charrel R, Coutard B, Eckerle I, Enouf V, Hungnes O, Korukluoglu G, Kossyvakis T, Mentis A, Molenkamp R, Muradrasoli S, Papa A, Pigny F, Thirion L, van der Werf S, Reusken C. 2020. Delayed laboratory response to COVID-19 caused by molecular diagnostic contamination. Emerg Infect Dis 26:1944–1946. doi:10.3201/eid2608.201843. 32433015 PMC7392437

[14] van Kasteren PB, van der Veer B, van den Brink S, Wijsman L, de Jonge J, van den Brandt A, Molenkamp R, Reusken C, Meijer A. 2020. Comparison of seven commercial RT-PCR diagnostic kits for COVID-19. J Clin Virol 128:104412. doi:10.1016/j.jcv.2020.104412. 32416600 PMC7206434

[15] Niedrig M, Linke S, Zeller H, Drosten C. 2006. First international proficiency study on West Nile virus molecular detection. Clin Chem 52:1851–1854. doi:10.1373/clinchem.2005.064451. 16887901 PMC7108183

[16] Pas SD, Patel P, Reusken C, Domingo C, Corman VM, Drosten C, Dijkman R, Thiel V, Nowotny N, Koopmans MP, Niedrig M. 2015. First international external quality assessment of molecular diagnostics for Mers-CoV. J Clin Virol 69:81–85. doi:10.1016/j.jcv.2015.05.022. 26209385 PMC7106520

[17] Lu X, Wang L, Sakthivel SK, Whitaker B, Murray J, Kamili S, Lynch B, Malapati L, Burke SA, Harcourt J, Tamin A, Thornburg NJ, Villanueva JM, Lindstrom S. 2020. US CDC real-time reverse transcription PCR panel for detection of severe acute respiratory syndrome coronavirus 2. Emerg Infect Dis 26:1654–1665. doi:10.3201/eid2608.201246. PMC7392423 32396505

[18] Institut Pasteur. 2020. Protocol: real-time RT-PCR assays for the detection of SARS-CoV-2. https://www.who.int/docs/default-source/coronaviruse/real-time-rt-pcr-assays-for-the-detection-of-sars-cov-2-institut-pasteur-paris.pdf?sfvrsn=3662fcb6_2. Institut Pasteur, Paris, France.

