# Supplementary material for: Variable Sensitivity of SARS-CoV-2 Molecular Detection in European Expert Laboratories: External Quality Assessment, June and July 2020
Source: J Clin Microbiol. 2021 Feb 18;59(3):e02676-20. doi: 10.1128/JCM.02676-20 (PMC8106723; doi:10.1128/JCM.02676-20)
Supplement: Supplemental file 1 [file JCM.02676-20-s0001.pdf]

Supplementary Table S1. Comparison of different extraction methods in EQA performance[a]

Extraction method	# Tested samples	Correct results
BioMérieux NucliSENS easyMAG	88	89.8%
EZ1 Virus Mini Kit v2.0	32	93.8%
MagMAX Viral/Pathogen Nucleic Acid Isolation Kit	33	90.9%
MagNA Pure Compact Nucleic Acid Isolation Kit I	33	87.9%
MagNA Pure 96 DNA and Viral NA SV Kit	44	90.9%
Roche Cobas Omni	55	89.1%
QIAamp Viral RNA Mini Kit	143	92.3%
QIAamp Viral RNA Mini QIAcube Kit	54	92.6%
Other method	286	89.4%

[a]None of the extraction methods performed significantly better than "Other methods" considering two-sided Yates' corrected chi square.
