# Supplementary material for: Variable Sensitivity of SARS-CoV-2 Molecular Detection in European Expert Laboratories: External Quality Assessment, June and July 2020
Source: J Clin Microbiol. 2021 Feb 18;59(3):e02676-20. doi: 10.1128/JCM.02676-20 (PMC8106723; doi:10.1128/JCM.02676-20)
Supplement: Supplemental file 2 [file JCM.02676-20-s0002.pdf]

Supplementary Table S2. List of all rRT-PCR methods that were used by EQA participants to analyse EQA panels.

**In house assays**	**Reference**	**Genome target**			
Corman *et al.*	(1)	E gene	N gene	RdRp gene 1	RdRP gene 2
US CDC, USA	(2)	N gene_N3	N gene_N1	N gene_N2	
Institute Pasteur, Paris, France	(3)	E gene	RdRp gene_IP2	RdRp gene_IP4	
HKU, Hong Kong SAR	(4)	Orf 1b			
China CDC, China	(5)	N gene			
not specified in house PCR	n.a.	n.a.			
**Commercial assays**					
**Company**	**Commercial kit**	**Genome target**			
altona Diagnostics GmbH	RealStar SARS-CoV-2 RT-PCR Kit 1.0	E gene	S gene		
AB Analytica s.r.l.	REALQUALITY RQ-2019-nCoV	RdRP gene	S gene		
Anchor Diagnostics GmbH	SARS-CoV-2 PCR Kit	not specified			
Bioeksen R & D Technologies Ltd.	Bio-Speedy SARS-CoV-2 (2019-nCoV) qPCR Detection Kit	RdRP gene			
Biohit HealthCare Ltd	IDK MutaPLEX Coronavirus Real-Time-RT-PCR-Kit	E gene	S gene	RdRP gene	
Bioneer Corporation	AccuPower SARS-CoV-2 Real-Time RT-PCR Kit	E gene	RdRP gene		
BioVendor - Laboratorni medicina a.s.	Liferiver Novel Coronavirus (2019-nCoV) Real Time Multiplex RT-PCR Kit	E gene	ORF1ab	N gene	
Cepheid	Xpert Xpress SARS-CoV-2	E gene	N gene (N2)		
CerTest Biotec	Viasure SARS-CoV-2 Real Time PCR Detection Kit	ORF1ab	N gene		
Co-Diagnostics, Inc.	Logix Smart COVID-19 Test (IVD)	RdRP gene			
Da An Gene Co., Ltd. of Sun Yat-sen University	Detection Kit 2019 Novel Coronavirus (2019-nCoV)	ORF1ab	N gene		
not specified	vDetect COVID-19 RT-qPCR	E gene	RdRP gene		
DiaSorin Molecular LLC	Simplexa COVID-19 Direct Kit	ORF1ab	S gene		
ELITechGroup	GeneFinder COVID-19 Plus RealAmp Kit	E gene	N gene	RdRp gene	
Euroimmun Medizinische Labordiagnostika AG	EURORealTime SARS-CoV-2	ORF1ab	N gene		
Fosun Pharma USA Inc.	Fosun COVID-19 RT-PCR Detection Kit	ORF1ab	N gene	E gene	
Integrated DNA Technologies, Inc.	2019-nCoV CDC EUA Kit	N gene_N1	N gene_N2		
Maccura Biotechnology Co., Ltd.	SARS-CoV-2 Fluorescent PCR Kit	ORF1ab	N gene	E gene	
NeuMoDx molecular	SARS-CoV-2 Assay	Nsp2 gene	N gene		
Primerdesign Ltd	Coronavirus COVID-19 genesig Real-Time PCR assay	not specified			
F. Hoffmann-La Roche Ltd	Cobas SARS-CoV-2 Test	E gene	ORF1ab		
Sacace Biotechnologies Srl	COVID-19 SARS-CoV-2 Real-TM	E gene	N gene		
SanSure Biotech Inc.	Novel Coronavirus (2019-nCoV) Nucleic Acid Diagnostic Kit	ORF1ab	N gene		
Seegene Inc.	Allplex 2019-nCoV Assay	E gene	RdRP gene	N gene	
Thermo Fisher Scientific Inc.	TaqPath COVID-19 CE-IVD RT-PCR Kit	ORF1ab	N gene	S gene	
TIB Molbiol GmbH	LightMix Modular SARS-CoV (COVID19) - RdRP	RdRP gene			
TIB Molbiol GmbH	LightMix Modular SARS-CoV (COVID19) - E-gene	E gene			

1. Corman VM, Landt O, Kaiser M, Molenkamp R, Meijer A, Chu DK, Bleicker T, Brunink S, Schneider J, Schmidt ML, Mulders DG, Haagmans BL, van der Veer B, van den Brink S, Wijsman L, Goderski G, Romette JL, Ellis J, Zambon M, Peiris M, Goossens H, Reusken C, Koopmans MP, Drosten C. 2020. Detection of 2019 novel coronavirus (2019-nCoV) by real-time RT-PCR. Euro Surveill 25.
2. Lu X, Wang L, Sakthivel SK, Whitaker B, Murray J, Kamili S, Lynch B, Malapati L, Burke SA, Harcourt J, Tamin A, Thornburg NJ, Villanueva JM, Lindstrom S. 2020. US CDC Real-Time Reverse Transcription PCR Panel for Detection of Severe Acute Respiratory Syndrome Coronavirus 2. Emerg Infect Dis 26.
3. Institut Pasteur P. 2020. Protocol: Real-time RT-PCR assays for the detection of SARS-CoV-2, *on* WHO. https://www.who.int/docs/default-source/coronaviruse/real-time-rt-pcr-assays-for-the-detection-of-sars-cov-2-institut-pasteur-paris.pdf?sfvrsn=3662fcb6_2. Accessed 26/11/2020.
4. Chu DKW, Pan Y, Cheng SMS, Hui KPY, Krishnan P, Liu Y, Ng DYM, Wan CKC, Yang P, Wang Q, Peiris M, Poon LLM. 2020. Molecular Diagnosis of a Novel Coronavirus (2019-nCoV) Causing an Outbreak of Pneumonia. Clin Chem 66:549-555.
5. Prevention CfDCa. Research use only 2019-novel coronavirus (2019-nCoV) real-time RT-PCR primers and probes (2020). http://ivdc.chinacdc.cn/kyjz/202001/t20200121_211337.html. Accessed 30/11/2020.
