# Supplementary material for: Variable Sensitivity of SARS-CoV-2 Molecular Detection in European Expert Laboratories: External Quality Assessment, June and July 2020
Source: J Clin Microbiol. 2021 Feb 18;59(3):e02676-20. doi: 10.1128/JCM.02676-20 (PMC8106723; doi:10.1128/JCM.02676-20)
Supplement: Supplemental file 3 [file JCM.02676-20-s0003.pdf]

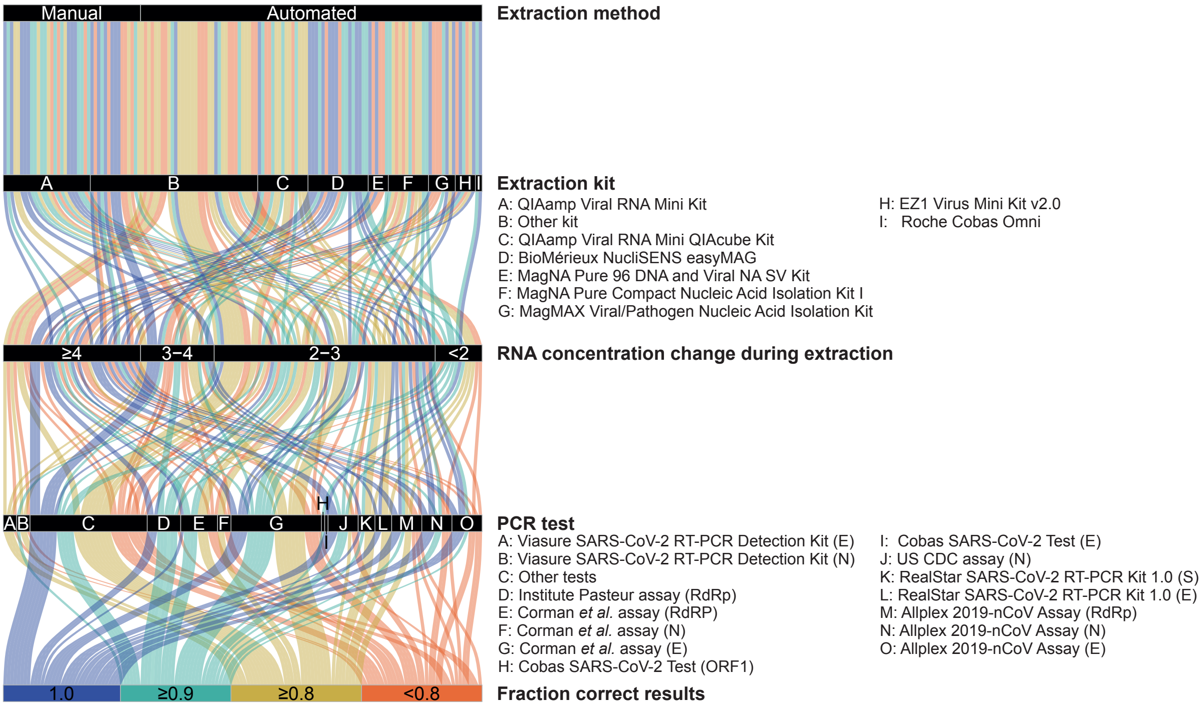

Supplementary Figure S1. Molecular workflow of all participating laboratories. Colours indicate EQA performance as fraction of correct results (1.0 = 100% correct, 0.8 = 80% correct): dark blue = 100% correct results, light blue = 90% correct results, yellow = 80% correct results and red = less than 80% correct results. No specific workflow combination, that is illustrated here as a combination of PCR, RNA concentration change during extraction, extraction kit and extraction method, correlates with a specific fraction of correct results, i.e. workflows that led to 100% correct results (all dark blue lines) included a wide variety of different PCR tests, various RNA concentration changes during extraction and numerous extraction kits. Only showing PCR tests that were used by at least 5 laboratories.
